# Clinical usefulness of the screen for cognitive impairment in psychiatry (SCIP-S) scale in patients with type I bipolar disorder

**DOI:** 10.1186/1477-7525-7-28

**Published:** 2009-04-01

**Authors:** Georgina Guilera, Oscar Pino, Juana Gómez-Benito, J Emilio Rojo, Eduard Vieta, Rafael Tabarés-Seisdedos, Nuria Segarra, Anabel Martínez-Arán, Manuel Franco, Manuel J Cuesta, Benedicto Crespo-Facorro, Miguel Bernardo, Scot E Purdon, Teresa Díez, Javier Rejas, the Spanish Working Group in Cognitive Function

**Affiliations:** 1 Department of Methodology, Faculty of Psychology, University of Barcelona, Barcelona, Spain; 2 Department of Psychiatry, Hospital General Granollers – Benito Menni CASM, Barcelona, Spain; 3 Bipolar Disorders Programme, Institute of Neuroscience, Hospital Clinic i Provincial, IDIBAPS, CIBER-SAM, University of Barcelona, Barcelona, Spain; 4 Teaching Unit of Psychiatry and Psychological Medicine, Department of Medicine, University of Valencia, CIBER-SAM, Valencia, Spain; 5 Programme Schizophrenia Clinic, Institute of Neuroscience, Hospital Clinic i Provincial, IDIBAPS, University of Barcelona, CIBER-SAM, Barcelona, Spain; 6 Department of Psychiatry, Hospital Provincial Rodríguez Chamorro, Zamora, Spain; 7 Psychiatric Hospitalization Unit, Hospital Virgen del Camino, Pamplona-Iruña, Spain; 8 Department of Psychiatry, Hospital University Marqués de Valdecilla, Santander, Spain; 9 Department of Psychiatry, Bebensee Schizophrenia Research Unit, University of Alberta, Edmonton, Alberta, Canada; 10 Department of Neurosciences, Medical Unit, Pfizer Spain, Alcobendas, Madrid, Spain; 11 Health Outcomes Research Department, Medical Unit, Pfizer Spain, Alcobendas, Madrid, Spain

## Abstract

**Background:**

The relevance of persistent cognitive deficits to the pathogenesis and prognosis of bipolar disorders (BD) is understudied, and its translation into clinical practice has been limited by the absence of brief methods assessing cognitive status in Psychiatry. This investigation assessed the psychometric properties of the Spanish version of the Screen for Cognitive Impairment in Psychiatry (SCIP-S) for the detection of cognitive impairment in BD.

**Methods:**

After short training, psychiatrists at 40 outpatient clinics administered the SCIP three times over two weeks to a total of 76 consecutive type I BD admissions. Experienced psychologists also administered a comprehensive battery of standard neuropsychological instruments to clinical sample and 45 healthy control subjects.

**Results:**

Feasibility was supported by a brief administration time (approximately 15 minutes) and minimal scoring errors. The reliability of the SCIP was confirmed by good equivalence of forms, acceptable stability (ICC range 0.59 to 0.87) and adequate internal consistency (Chronbach's alpha of 0.74). Construct validity was granted by extraction of a single factor (accounting 52% of the variance), acceptable correlations with conventional neuropsychological instruments, and a clear differentiation between bipolar I and normal samples. Efficiency was also provided by the adequate sensitivity and specificity.

**Limitations:**

The sample size is not very large. The SCIP and the neurocognitive battery do not cover all potentially relevant cognitive domains. Also, sensitivity to change remains unexplored.

**Conclusion:**

With minimal training, physicians obtained a reliable and valid estimate of cognitive impairment in approximately 15 minutes from an application of the SCIP to type I BD patients.

## Background

Cognitive deficits in bipolar disorders are relevant to cerebral pathogenesis and prognosis, but they are often neglected in routine clinical practice. The deficits persist beyond the resolution of acute symptoms [1-3] and show familial co-segregation [4] consistent with expectations for a genetically based endophenotypic trait [5]. The cognitive deficits in bipolar disorder are also directly related to functional status or psychosocial outcomes [6,7], and the severity of the cognitive impairment at initiation of therapeutic intervention can be a powerful predictor of functional recovery one year later [8]. Similar observations in schizophrenia [9] prompted the National Institutes of Health initiative for Measurement and Treatment of Cognitive Impairment in Schizophrenia [10], and a parallel initiative for bipolar disorder may be in order. This would be facilitated by a more wide spread incorporation of cognitive assessments into the routine clinical examinations of bipolar patients.

A relative paucity of routine cognitive assessments in current clinical practice is apparent, however, and this may relate to a perceived absence of feasible and valid methods of quantification. For example, although the Repeatable Battery for the Assessment of Neuropsychological Status (RBANS) [11], and the Brief Assessment of Cognition in Schizophrenia (BACS) [12], are useful tools for psychiatric patients, they require relatively expensive test-administration kits and at least 30 minutes for administration. In contrast, the Mini-Mental State Examination (MMSE) [13], a staple in the assessment of cognitive deficits associated with neurological disorders, is completed on a single printed page of paper in approximately 15 minutes. Although the MMSE is sensitive to the severity of dementia in geriatric samples, it has proven to be unstable and unreliable in psychotic or affective disorders where it underestimates the cognitive disturbance in younger samples and overestimates pathology in older, less educated, or less intelligent samples [14-17].

The Screen for Cognitive Impairment for Psychiatry (SCIP) [18] was developed to offer a brief tool for the quantification of cognitive deficits in higher functioning psychiatric patients. The SCIP consists of a single page with five subtests of cognitive skill that can be completed with a pencil and a timer. Each subtest requires two to three minutes, for a total administration time of approximately 15 minutes. The subtests within the SCIP quantify working memory, immediate and delayed verbal list learning, verbal fluency, and psychomotor speed, all of which may be impaired in schizophrenia or bipolar disorders [3,19-22]. Three alternate forms of the SCIP are available, with good reliability, validity, and form equivalence [18]. A direct translation to Spanish, with minor changes from the English variant, was accomplished by two Spanish-born bilingual professionals, one a Spanish philology graduate with extensive knowledge of English, and the second a neuropsychologist with expertise in the scale contents. A back translation was deemed an adequate representation of the original English version after it was created by a native English-speaking bilingual language school teacher from the University of Barcelona, who also possessed a degree in psychology. The three alternate forms of the Spanish-language SCIP (SCIP-S) demonstrated equivalence, reliability, and validity in a University-recruited normal control sample [23]; the sensitivity, reliability, and validity of the SCIP-S was recently confirmed in a large sample of patients suffering from schizophrenia [24]. The present investigation was designed to assess the sensitivity, reliability and validity of the SCIP for detection and quantification of cognitive impairment in euthymic patients suffering from a type I bipolar disorder.

## Method

### Sample

Participants included a sample of 76 bipolar I patients and a sample of 45 matched healthy controls, all of whom provided written consent after full disclosure of the study methods. The clinical sample was recruited through 40 outpatient psychiatric clinics across Spain, so the sample selection had a nested structure. They were 18 to 55 years of age with a type I bipolar disorder diagnosed by an experienced psychiatrist according to DSM IV-TR criteria [25]. They were in a stable phase of the illness defined by at least 6 months in remission, a Hamilton Depression Scale (HAMD) [26] score less than 8, a Young Mania Rating Scale (YMRS) [27] score less than 6, and no required changes in the type or dose of psychopharmacological treatment for the duration of the study. Subjects with severe or unstable medical or neurological problems, illiterate, other primary psychiatric disorders including major depression, or ongoing participation in a clinical trial were excluded. The control sample was statistically matched to the clinical sample on sex, age, and educational level, and they were free of significant symptoms of psychiatric illness assessed with the interview Comprehensive Assessment of Symptoms and History (CASH) [28]. Controls were excluded if they had severe medical or neurological problems, met criteria for a psychiatric disorder, were participating in a clinical trial, were illiterate, or having any first degree relative with mental illness.

### Procedure

The method was reviewed and approved by the Ethics Committee of the University of Barcelona. The bipolar sample participated in three test sessions, denoted below as Visits 1 (V1), 2 (V2), and 3 (V3), whereas the control sample participated in only one test session at V1. The baseline V1 session for all subjects consisted of an interview to obtain sociodemographic background, a SCIP examination, and approximately 1.5 hours of testing with conventional standardized neuropsychological instruments (NPS). The CASH was also completed with the control subjects at V1. The SCIP was administered by one of 44 psychiatrists, and the NPS examination was administered by one of 41 neuropsychologists. The psychiatrists participated in a one hour SCIP training session provided by a neuropsychologist with extensive experience in cognitive assessment and four years' experience with the SCIP. The baseline (V1) NPS examination consisted of the Edinburgh Handedness Inventory [29], the Wechsler Adult Intelligence Scale-III (WAIS-III) [30], Vocabulary, Symbol Search, Digit-Symbol Coding, Arithmetic, Digit Span, and Letter-Number Sequencing Subtests, as well as the Wechsler Memory Scale-III (WMS-III) [31], Wordlist I and Wordlist II Subtests, the Wisconsin Card Sorting Test (WCST) [32], the Trail Making Test (TMT- A-B) [33] the Semantic Fluency Test [34,35]. The bipolar sample also participated in a clinical assessment at baseline (V1) that was repeated seven (V2) and fourteen (V3) days later and included ratings on the YMRS, the HAMD, the Clinical Global Impression inventory (CGI-G) [36], the Social and Occupational Functioning Assessment Schedule (SOFAS-EEASL) [37], and the SCIP. Participants were randomly assigned to receive one of six combinations of SCIP form orders constructed from a complete counterbalance of alternate forms between V1 and V2 to assess practice effects, followed by a repetition of the V2 form at V3 to assess common form stability (i.e., forms 1-2-2, 1-3-3, 2-1-1, 2-3-3, 3-1-1, 3-2-2). Additional details of the instrumentation for quantification of history, psychosocial status, and clinical symptoms, as well as the measures applied in the standardized NPS screen are widely referenced and available in their respective manuals.

Each of the three alternate forms of the SCIP [18] contains an equivalent Verbal Learning Test with Immediate Recall (VLT-I), a Working Memory Test (WMT), a Verbal Fluency Test (VFT), a Verbal Learning Test with Delayed Recall (VLT-D), and a Processing Speed Test (PST). The VLT-I is a variant of the Rey Auditory Verbal List Learning Test (RAVLT) [38] consisting of three trials of a 10 word list-learning task with immediate recall after each spoken presentation of the list. The primary dependent variable is the sum of the number of words correctly recalled over the three trials. The WMT is a variant of the Brown-Peterson Consonant Trigram Test (CTT) [39,40], consisting of eight 3-letter combinations of consonants, with two trigrams each assigned to a 0, 3, 9, or 18 second delay with backward counting distraction. The primary dependent variable is the sum of the letters correctly recalled. The VFT is a variant of the Controlled Oral Word Association Test (COWAT) [41] consisting of two trials of 30 seconds during which the subject is invited to generate words that begin with a given letter of the alphabet while avoiding numbers, proprietary names, or a single root with multiple suffixes. The dependent variable is the sum of acceptable words over the two trials. The VLT-D consists of a delayed recall test of the VLT-I words. The PST is an original visuomotor tracking task that requires the subject to translate the Morse code equivalents of six letters from the alphabet in boxes under a randomly distributed sequence of the letters. The dependent variable consists of the number of correct sequential translations in 30 seconds.

### Statistical analysis

The feasibility of the SCIP was examined in relation to the scoring and correction errors, patient tolerance of the procedures, and the time required to complete the SCIP.

The reliability of the SCIP was assessed by examination of alternate form equivalence, the test-retest reliability, the internal consistency within the bipolar sample, and the magnitude of practice effects observed with alternate forms. The alternate form equivalence was measured by means of a multivariate and univariate analysis of variance of baseline subtest and total scores, respectively. Test-retest reliability was assessed with intra-class correlation coefficients (ICC) between the first and second administrations of common forms (i.e. V2/V3). Consistency was examined using Cronbach's alpha coefficient applied to the three SCIP administrations. Reliability was also assessed by multivariate (subscale scores) and univariate (total score) analysis of practice effects at baseline (V1) and V2; the magnitude of these practice effects was calculated with Cohen's *d*, representing the difference between mean scores at V2 and baseline divided by the standard deviation of the baseline visit.

The validity of the SCIP for a quantification of cognitive impairment in bipolar disorder was examined with an assessment of construct and convergent validity, and scale sensitivity and specificity. Construct validity was examined by principal components factor analysis of the SCIP subscale scores. Correlations between subtests were evaluated by means of Pearson correlation coefficients. The relations between total SCIP score and neuropsychological battery were also explored by means of Pearson correlation coefficient. The validity of the SCIP in the differentiation between the bipolars and matched healthy control sample was assessed by multivariate and univariate comparison of the baseline SCIP subscale scores and total score, respectively. The magnitude of these differences on each subscale score, and the total score, was calculated with Cohen's *d*, representing the difference between the baseline mean scores of controls and patients divided by the pooled standard deviation. Also, the ability of the SCIP to distinguish between patients who had cognitive impairment and those who did not, was assessed carrying out a sensitivity and specificity analysis of the SCIP total score in the framework of logistic regression and receiver operating curve (ROC) analysis. The criterion to establish the differentiation between cognitive impairment and non-cognitive impairment in the bipolar sample was based on a global weighted *z *score of the neuropsychological battery (excluding the SCIP) after standardization using the baseline control group. Several studies have confirmed that cognitive impairment severity in patients with bipolar disorder is about 0.5 to 1.5 standard deviations lower than the healthy population [42,43]; consequently as a general criterion the cut off score was established at ≤ -1 standard deviation.

The SPSS version 12.0 statistical software was used with significance levels of 0.01 for Pearson correlations and 0.05 in all other cases.

## Results

### 1. Sample descriptive statistics

The bipolar I sample consisted of 76 patients with a mean duration of illness of 146.88 (SD = 96.96) months. They had experienced an average of 4.38 (SD = 3.35) manic episodes, 4.33 (SD = 4.47) depressive episodes, and 3.31 (SD = 4.31) hospital admissions. Comorbid disorders were apparent in two patients (2.63%) included in the present bipolar I sample and included one patient with obsessive compulsive features and another with anxiety features. At the time of cognitive assessment, patients were on lithium (N = 22; 28.85%), lithium plus one antipsychotic (N = 31; 40.79%), lithium plus two antipsychotics (N = 3; 3.95%), one antipsychotic medication (N = 12; 15.79%), two antipsychotics (N = 2; 2.63%) or three antipsychotics (N = 1; 1.32%). Five patients were free of lithium and antipsychotics. Some patients (N = 52; 68.42%) were additionally taking another drug treatment (e.g. antidepressants, benzodiazepines). All patients were maintained on their baseline medications without modifications of dose through the course of this investigation. Caffeine was consumed within 24 hours prior to testing by 52.6% of the sample, nicotine by 53.9%, and alcohol by 1.3%. The principal sociodemographic variables of the study sample are reported in Table 1. On the baseline examination, the bipolar group received average SOFAS ratings at of 76.81 (SD = 14.27), midway between "some difficulty" and "good functioning".

**Table 1:** Demographic characteristics of the study sample.

**Variable**	**N**	**%**	**Variable**	**N**	**%**
Sex			Cohabitation		
Male	34	44.7	Family of origin	30	39.5
Female	42	55.3	Own family	35	46.1
Marital status			Friends	0	0
Single	35	46.1	Tutelage home	0	0
Married	25	32.9	Alone	9	11.8
Living as couple	6	7.9	Others	2	2.6
Widowed	1	1.3	Occupational status		
Separated/divorced	9	11.8	Student	2	2.6
Educational level			Employee	21	27.6
Illiterate	0	0.0	Liberal professional	8	10.5
Functionally illiterate	1	1.3	Non-remunerated work	1	1.3
Primary	28	36.8	Disabled	20	26.3
Secondary	24	31.6	Unemployed	8	10.5
University studies	22	28.9	Retired	8	10.5
Others	1	1.3	Housewife	8	10.5
Age *Mean (SD)*	76	40.30 (8.98)			

The control group was composed of 25 males and 20 females with a mean age of 37.69 (SD = 8.20) ranging from 20 to 54 years old. Regarding their education level, 31.10% followed primary education, 40.00% secondary education and 28.90% had university education. No significant differences were observed between the patient and control groups in gender (χ^2^_(1) _= 1.324; p = 0.250), education (χ^2^_(4) _= 2.03; p = 0.730), or age (t_(119) _= 1.597; p = 0.113).

### 2. Feasibility

The relatively brief training session for the SCIP administration resulted in a mean kappa index of agreement in scale correction and scoring of 0.99. The SCIP was tolerated well by both bipolar patients and normal controls, as apparent in the successful completion of a complete baseline SCIP in all 76 bipolar patients and 45 controls. Seventy-four patients (97.37%) reported to all three programmed visits. One bipolar subject completed the SCIP at all three sessions but refused to complete some of the baseline NPS tests. Two bipolar subjects did not return for the third session and gave no explanation for their discontinuation. The average time to complete each of the first two SCIP assessments in the bipolar sample was 15.61 (SD = 5.01) and 14.16 (SD = 4.92) minutes.

### 3. Reliability

The equivalence of the three alternate forms of the SCIP was supported by the absence of significant differences between forms in a multivariate analysis of variance comparing the baseline scores of the bipolar sample, (F_(10,140) _= 0.813, p = 0.616) (see Table 2). Univariate comparisons also revealed no significant differences between the alternate subtests of the SCIP (all p > 0.05).

**Table 2:** Mean SCIP scores for each of the parallel forms.

	**Form 1**		**Form 2**		**Form 3**			
**Subtest**	**N**	**Mean (SD)**	**N**	**Mean (SD)**	**N**	**Mean (SD)**	**Significance**	

VLT-I	25	19.08 (4.50)	24	20.50 (4.72)	27	18.70 (3.85)	F_(2,73) _= 1.179	p = 0.313
WMT	25	17.16 (3.64)	24	17.71 (3.99)	27	17.04 (4.11)	F_(2,73) _= 0.206	p = 0.814
VFT	25	14.64 (5.31)	24	15.96 (5.87)	27	12.85 (4.50)	F_(2,73) _= 2.272	p = 0.110
VLT-D	25	5.36 (2.69)	24	5.54 (2.60)	27	4.93 (2.00)	F_(2,73) _= 0.435	p = 0.649
PST	25	9.44 (2.22)	24	8.75 (2.74)	27	9.11 (3.18)	F_(2,73) _= 0.385	p = 0.682

Total SCIP	25	65.68 (13.71)	24	68.46 (13.92)	27	62.63 (13.65)	F_(2,73) _= 1.167	p = 0.317

VLT-I = Verbal Learning Test-Immediate; WMT = Working Memory Test; VFT = Verbal Fluency Test; VLT-D = Verbal Learning Test-Delayed; PST = Processing Speed Test; Total SCIP = SCIP total score

Good test-retest reliability was also apparent in the intra-class correlation coefficients (ICC) between the first and second administrations of common forms (i.e. V2/V3), with values ranging from a low of 0.59 for the VLT-D and a high of 0.82 for the PST (see Table 3). The sum of the subscale scores achieved an ICC of 0.87.

**Table 3:** SCIP Intra-Class Correlation Coefficients (ICC).

	**Visit 2 (V2)**		**Visit 3 (V3)**		
**Subtest**	**N**	**Mean (SD)**	**N**	**Mean (SD)**	**ICC**

VLT-I	74	19.95 (3.81)	74	22.11 (4.07)	0.75
WMT	74	17.70 (4.18)	74	18.11 (4.18)	0.77
VFT	74	15.09 (4.86)	74	16.03 (5.27)	0.67
VLT-D	74	5.23 (2.14)	74	6.62 (2.06)	0.59
PST	74	9.45 (3.13)	74	9.95 (3.31)	0.82

Total SCIP	74	67.42 (13.74)	74	72.81 (14.17)	0.87

VLT-I = Verbal Learning Test-Immediate; WMT = Working Memory Test; VFT = Verbal Fluency Test; VLT-D = Verbal Learning Test-Delayed; PST = Processing Speed Test; Total SCIP = SCIP total score

The SCIP subtests exhibited adequate internal consistency, with Chronbach's alphas of 0.74, 0.78, and 0.77 at Visits 1, 2 and 3, respectively.

The multivariate analysis of variance suggested the absence of a general practice effect between alternate forms administered at baseline (V1) and one week later (V2) (F_(5,70) _= 2.20, p = 0.064), but univariate comparison between V1 and V2 for total SCIP score revealed significant differences (see Table 4). This contrasts with the multivariate comparison between common forms administered at V2 and one week later (V3) (F_(5,69) _= 13.47, p < 0.05), resulting from significant improvements on the second administration of all subscales (p < 0.05) except the WMT (t_(73) _= 1.23, p = 0.221) and the VFT (t_(73) _= 1.95, p = 0.055).

**Table 4:** Mean SCIP scores on the baseline (V1) and one week later (V2).

	**Baseline (V1)**		**Visit 2 (V2)**				
**Subtest**	**N**	**Mean (SD)**	**N**	**Mean (SD)**	**Significance**		**Cohen d**

VLT-I	75	19.41 (4.39)	75	19.91 (3.80)	t_(74) _= 1.074	p = 0.286	0.11
WMT	75	17.25 (3.89)	75	17.73 (4.16)	t_(74) _= 1.558	p = 0.123	0.12
VFT	75	14.43 (5.35)	75	15.16 (4.86)	t_(74) _= 1.766	p = 0.081	0.14
VLT-D	75	5.28 (2.43)	75	5.23 (2.13)	t_(74) _= 0.214	p = 0.831	-0.02
PST	75	9.12 (2.75)	75	9.48 (3.12)	t_(74) _= 1.628	p = 0.108	0.13

Total SCIP	75	65.49 (13.74)	75	67.51 (13.67)	t_(74) _= 2.231	p = 0.029*	0.15

* p < 0.05

VLT-I = Verbal Learning Test-Immediate; WMT = Working Memory Test; VFT = Verbal Fluency Test; VLT-D = Verbal Learning Test-Delayed; PST = Processing Speed Test; Total SCIP = SCIP total score

### 4. Validity evidences

The construct validity of the SCIP as a measure of cognitive impairment in bipolar disorder was supported by the principal component factor analysis extraction of a single factor with an eigenvalue of 2.610 accounting for 52.21% of total variance, and the high loading of each of the five subtests on this factor, ranging from 0.53 for the PST to 0.81 for VLT-D. Moreover, the analysis of internal consistency by means of the Cronbach's alpha coefficient showed that removal of any subtest would involve a decrease in this coefficient, which supports the relevance of each of them. The correlations between SCIP subtests' scores are shown in table 5; they range from 0.18 (VLT-I – PST) to 0.68 (VLT-I – VLT-D).

**Table 5:** Correlations between SCIP subtests.

**Subtest**	VLT-I	WMT	VFT	VLT-D	PST
VLT-I	-	0.38*	0.43*	0.68*	0.18
WMT		-	0.44*	0.41*	0.36*
VFT			-	0.47*	0.36*
VLT-D				-	0.24
PST					-

* p < 0.01

VLT-I = Verbal Learning Test-Immediate; WMT = Working Memory Test; VFT = Verbal Fluency Test; VLT-D = Verbal Learning Test-Delayed; PST = Processing Speed Test; Total SCIP = SCIP total score

Pearson's correlation coefficient between the SCIP total score and the global weighted z score from the neuropsychological battery abovementioned was 0.74 (p < 0.01), suggesting that the SCIP provides valid measure of global cognitive impairment in bipolar disorder. A significant correlation was also observed between SCIP total score and the SOFAS (r = 0.45, p < 0.01).

In relation to differentiation between patients and controls, the multivariate analysis revealed a significant difference between groups (F(5,115) = 11.28, p < 0.05), with the healthy controls exceeding the performance of the bipolar group on all five SCIP subtests (all p < 0.05), with Cohen d effect sizes ranging from a medium on the two VLT and WMT subtests to a high on the other subtests (see Table 6). The univariate comparison of the total SCIP score also revealed a high effect size (d = 1.16).

**Table 6:** Mean SCIP scores on the baseline visit (V1).

	**Patients**			**Controls**			
**Subtest**	**N**	**Mean (SD)**	**Min. – Max.**	**N**	**Mean (SD)**	**Min. – Max.**	**Cohen d**

VLT-I	76	19.39 (4.36)	9 – 28	45	21.84 (3.74)	14 – 29	0.59
WMT	76	17.29 (3.88)	8 – 24	45	19.58 (3.17)	12 – 24	0.63
VFT	76	14.42 (5.32)	4 – 31	45	19.73 (5.71)	6 – 32	0.97
VLT-D	76	5.26 (2.42)	0 – 10	45	6.69 (1.87)	2 – 10	0.64
PST	76	9.11 (2.73)	2 – 14	45	12.49 (2.72)	5 – 18	1.24

Total SCIP	76	65.47 (13.57)	27 – 93	45	80.33 (11.06)	40 – 101	1.16

VLT-I = Verbal Learning Test-Immediate; WMT = Working Memory Test; VFT = Verbal Fluency Test; VLT-D = Verbal Learning Test-Delayed; PST = Processing Speed Test; Total SCIP = SCIP total score

**Figure 1:**
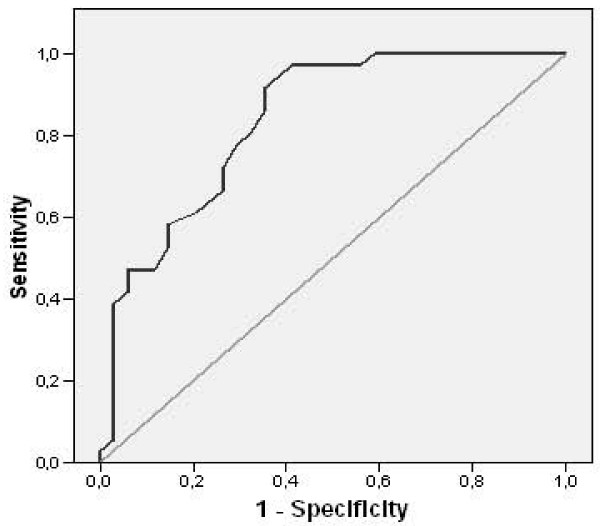
**ROC curve of the differentiation between patients with and without cognitive impairment**.

Moreover, in order to assess the ability of the SCIP to distinguish between patients who had cognitive impairment and those who did not, logistic regression analysis was performed. The SCIP total score coefficient was significant as a classification variable, presenting a global percentage of correct classifications of 74.28%, a Nagelkerke R^2 ^of 0.453, and the Hosmer-Lemeshow statistics was no significant. The ROC analysis obtained an area under the curve of 0.837 (p < 0.05) (see Figure 1). The most balanced cut-off point was established at ≤ 67, presenting a sensitivity of 73.53%, and a specificity of 72.22%.

## Discussion

The well documented cognitive limitations associated with bipolar disorders [3,19] are recognized by the patients [44], have characteristics of an illness endophenotype [4], and directly relate to functional or psychosocial recovery after the onset of illness [6,8,45]. Despite the apparent relevance of cognitive impairment to diagnosis, pathogenesis, and prognosis, this aspect of the illness is often neglected in both clinical practice and epidemiological studies [46]. The neglect may relate to the lack of brief standardized instruments with proven validity for the detection and quantification of the cognitive limitations associated to bipolar disorders. The SCIP [18] was developed to address several limitations of similar tools, and the current prospective evaluation provides the first demonstration supporting the feasibility, reliability, and validity of the Spanish version of the SCIP (SCIP-S) in a bipolar sample. The feasibility of the SCIP-S for routine clinical practice was supported by minimal errors of implementation or scoring committed by psychiatrists after a relatively brief one hour training session, a brief administration time of approximately 15 minutes per patient, and the minimal instrument requirements (paper, pencil, and clock). Tolerability was also demonstrated.

In addition to feasibility and tolerability, the current investigation supported the reliability and the validity of the SCIP applied to bipolar disorder. The three alternate forms of the SCIP-S produced equivalent baseline scores on all five subtests, and the overall intra-class coefficient was high. Very similar reliability results have been reported in normal college student samples for both the original English SCIP [18] and the Spanish SCIP [24]. The construct validity of the SCIP for detection of cognitive impairment in bipolar disorder was supported by the extraction of a single significant factor, namely cognitive impairment, and the high correlation between the neuropsychological battery and the overall factor score. Similar results were encountered with a larger schizophrenic sample, where the scores also converged on a single cognitive factor accounting for almost 50% of the total variance [24]. The SCIP general score was also correlated with functional status, as anticipated from prior studies with more prolonged cognitive batteries. As confirmed by sensitivity and specificity analysis, and taking into account that the SCIP is a screening test, the SCIP total score is an acceptable measure for distinguishing between patients with and without cognitive impairment.

In sum, the SCIP produced a valid quantification of cognitive status in bipolar patients by psychiatrists with minimal training on the tool, and the small magnitude of practice effects with the alternate forms suggest that it may also be useful for monitoring progress through time.

### Limitations and future research

The present results offer support for the feasibility, reliability, and validity of the SCIP as a tool for screening cognitive impairment in bipolar disorders, but some limitations should be taken into account. A major limitation of the present study refers to the sample size, which is in the usual range of this sort of studies and provides a first step towards future investigations of the clinical applications of this tool, but does not allow more sophisticated statistical subanalysis. Additional data will be required to confirm the value of the scale in larger clinical samples.

Another potential weakness relates to the election of the neuropsychological battery. It should be taken into account that any effort to validate the SCIP with a gold standard battery will be limited by the validity, reliability, and adequacy of the battery.

The SCIP is missing coverage of several potentially important cognitive domains that are likely relevant to bipolar disorder, including measurement of executive function and nonverbal skills. Supplemental tests will be required in situations where these skills are a priority. However, we are also investigating supplemental scoring strategies, including errors of intrusion and perseverations on the verbal fluency and verbal learning subtests of the SCIP; these additional scores may offer valuable measures of frontal lobe functions that should correlate with executive and problem solving skills.

Another priority of our future research will be trying to make a link between individual measures on the SCIP and corresponding individual measures on the larger neuropsychological battery, in order to explore if the SCIP subscale scores provide valid measures of their underlying cognitive domains in bipolar disorder.

The SCIP seems to work properly when it is administered by psychiatrists who have participated in a relatively brief training exercise. In future investigations it would be useful to train and evaluate other mental health care professionals, including nurses, occupational therapists, and social workers, all of whom are likely equipped with the skills necessary for a reliable and valid implementation of this relatively simple standardized assessment instrument. The SCIP will not replace the diagnostic value of a full neuropsychological examination, but it will offer a rapid inexpensive mechanism for screening cases with a lower probability of significant impairments. In future investigations it would be useful to evaluate the relative sensitivity and specificity of the SCIP against structural and functional in vivo neuroimaging evidence of cerebral pathology in psychiatric and neurological disorders. There is also fairly broad agreement that cognitive limitations are related to the educational, occupational, and social limitations that result from bipolar disorders, and it would be useful to assess the prognostic value of the SCIP to psychosocial outcome in future prospective investigations. It would be also interesting to explore SCIP scores in patients with bipolar disorder compared to other psychiatric disorders; in this sense preliminary results comparing bipolar and schizophrenic samples have shown slight differences with the bipolar patients exceeding the performance of the schizophrenic patients on all five SCIP subtests, but with low *d *effect sizes ranging from d = 0.00 for PST to 0.30 for VLT-D.

## Conclusion

Finally, the brevity of the SCIP underscores its potential value to clinical trials with bipolar disorders directed toward an improvement in cognitive skills that may mitigate an improvement in functional outcome. The current investigation supported the use of alternate forms to reduce practice effects, potentially increasing sensitivity to treatment-related changes, but the sensitivity of the SCIP to pharmacotherapeutic interventions has yet to be confirmed.

## Competing interests

This study was financed by Pfizer Spain and supported by projects 2007FIC00736, and 2005SGR00365 of the "Departament d'Universitats, Recerca i Societat de la Informació de la Generalitat de Catalunya", and SEJ2005-09144-C02-02/PSIC of the "Ministerio de Educación y Ciencia de España". This study was also supported by the Spanish Ministry of Health, Instituto de Salud Carlos III, CIBER de Salud Mental (CIBER-SAM).

Javier Rejas is employed by Pfizer Spain. Teresa Díez was employed by Pfizer at the time of conduction of study. All other authors declare that they have no conflicts of interest related to this study.

## Authors' contributions

GG, OP, JG, JER, EV, TD, JR conceived of the study and participated in its design, coordination and analysis of the results as well as helped to draft the manuscript. RT, NS, AMA, MF, MC, BC, MB, SP participated in the patients' recruitment as well as helped to design and draft the manuscript.

## Appendix

In addition to the authors, the following were members of the SCIP study collaborative group: J Aguilar, ASM Puzol, Valencia; C Aguirre, Hospital Santa Eulalia, Barcelona; M Alcañiz, CSM Alcobendas, Madrid; R Alarcón, CSM de Cartagena, Murcia; JP Alcón, ESM Oriente, Sevilla; MM Alda, USM de Alcañiz, Zaragoza; M Alonso, CSM de Torrelavega, Torrelavega; B Alvarez del manzano, CSM de Retiro, Madrid; V Balanza, USM de Catarroja, Valencia; MT Bel Villar, CSM de Mollet, Barcelona; P Benavent, Hospital Universitario La Fe, Valencia; JC Berenguer, Hospital Universitario Ntra Sra de la Candelaria, Santa Cruz de Tenerife; AI Bernal, Hospital de Valme, Sevilla; AL Blanco, CS Provincial de Plasencia, Cáceres; Y Bueno, Complejo asistencial de Zamora, Valladolid; J Calvo, Hospital Santa Maria, Tarragona; M Camacho, CSM Macarena Sevilla; S Campanera, CSM de Lleida, Lleida; S Campanera, Hospital Santa María, Tarragona; M Campillo, Hospital Morales Meseguer, Murcia; A Carrillo, CSM Moratalaz, Madrid; S Cesteros, Hospital Morales Meseguer, Murcia; D Closas, CSM dreta de l'eixample, Barcelona; C Conesa, CSM Mollet, Barcelona; FJ Cotobal, CSM Arganda, Madrid; L Chamorro, Hospital General Universitario Guadalajara, Madrid; A Deu Coll, CSM Santa Coloma de Farners, Girona; P Ecenarro, CSM Fontiñas, La Coruña; G Faus, CSM dreta de l'eixample, Barcelona; JL Fernández, USM Canalejas, Las Palmas; M Franco, Complejo asistencial de Zamora, Zamora; A Fuentes, Hospital Ingesa, Ceuta; C García, CSM Las Torres, Burgos; MJ García-Pereda, CS Provincial de Plasencia, Cáceres; MP Garcia-Portilla, Facultad de Medicina de Oviedo, Asturias; LF Gaton, Hospital Ingesa, Ceuta; JM Goicolea, Hospital Clinic de Barcelona, Barcelona; MJ González, CSM de Hortaleza, Madrid; MP González, CSM de Lleida, Lleida; S González, Facultad de Medicina de Oviedo, Asturias; S González, Hospital de Valme, Sevilla; C González de Vega, CSM de Hortaleza, Madrid; P Iborra, CSM Cabo Huerta, Alicante; J Latorre, Hospital Santa Eulalia, Barcelona; C Lorenzo, CSM Fontiñas, La Coruña; L Luna, Hospital Universitario La Fe, Valencia; P Luna, Fundacion Argibide, Navarra; A Mane, Hospital Clinic, Barcelona; I Mata, Fundacion Argibide, Navarra; V Martí, CSM de Paterna, Valencia; F Martín, CSM Las Torres, Burgos; A Martínez-Arán, Hospital Clinic, Barcelona; JP Martínez, CSM de Cartagena, Murcia; M Martínez, CSM Actur Sur, Zaragoza; R Martínez, Esma-Loja, Granada; S Martínez, Hospital Clinic, Barcelona; B de Mazarrasa, Hospital General Universitario de Guadalajara, Guadalajara; F Megias del Rosal, USM Puzol, Valencia; E Melo, CSM Cabo Huerta, Alicante; J Merino, CSM Santa Coloma de Farners, Girona; JM Misiego, Hospital Son Llatzer, Palma de Mallorca; O Vallina, SCM de Torrelavega, Torrelavega; JA Ortega, USM de Alcañiz, Zaragoza; A Pascual, USM de Alcañiz, Zaragoza; J Pérez, CSM Alcobendas, Madrid; MT Pérez, Hospital Universitario Ntra Sra de la Candelaria, Santa Cruz de Tenerife; J Ponte, Hospital de Zamudio, Vizcaya; M Reyes, Esma-Loja, Granada; JM Rodríguez, USM Puertochico, Cantabria; R Romero, ESM Oriente, Sevilla; C Rubio, USM de Catarroja, Valencia; G Rubio, CSM de Retiro, Madrid; FC Ruiz, Hospital Río Carrión, Palencia; G Safon, Cap Rambla, Barcelona; J Salazar, CSM de Paterna, Valencia; R Sanguino, Hospital Río Carrión, Palencia; M Santoja, Cap Rambla, Barcelona; N Segarra, Hospital Clinic, Barcelona; MJ Serrano, Hospital Son Llatzer, Palma de Mallorca; D Sierra, USM Puertochico, Cantabria; AB Tejero, Centro de Salud Mental de Cartagena, Murcia; S Torrijos, CSM Moratalaz, Madrid; JJ Uriarte, Hospital de Zamudio, Vizcaya; A Vallespi, CSM Actur Sur, Zaragoza; N Valverde, CSM Arganda, Madrid; JA de Vega, USM Canalejas, Las Palmas.
